# Effect of Time Interval From Diagnosis to Treatment on Economic Burden in Families of Children With Autism Spectrum Disorder

**DOI:** 10.3389/fpsyt.2021.679542

**Published:** 2021-11-26

**Authors:** Wensu Zhou, Kui Wu, Shu Chen, Dan Liu, Huilan Xu, Xiyue Xiong

**Affiliations:** ^1^Department of Social Medicine and Health Management, Xiangya School of Public Health, Central South University, Changsha, China; ^2^Department of Child Rehabilitation, Hunan Provincial Maternal and Child Health Care Hospital, Changsha, China; ^3^NHC Key Laboratory of Birth Defect for Research and Prevention, Hunan Provincial Maternal and Child Health Care Hospital, Changsha, China

**Keywords:** autism spectrum disorder, economic burden, family, China, time interval

## Abstract

The study aimed to investigate the economic costs in families of children with autism spectrum disorder (ASD) and explored how the time interval from diagnosis to treatment time interval from the date children first diagnosed with ASD to the date of first speech/behavior treatment influenced the economic costs. It was a cross-sectional study that recruited families with autistic children in Changsha, Hunan Province of China during March to November 2018. A self-designed questionnaire was applied to collect ASD-related economic costs in the two vital periods including the 12-month period after diagnosis and the most recent 12 months during the course of ASD. In total, 136 families with autistic children completed the interview. The results showed that 46.3% of children started intervention within 1 month. The median of total economic costs of these families in the 12-month period after diagnosis and the most recent 12 months was 26,502.26 RMB and 29,411.91 RMB, respectively. Compared with the time interval shorter than 1 month, time interval over 6 months was significantly associated with high direct economic costs (β_*SD*_ = 0.308, 95% CI = 0.177, 1.254), inpatient/outpatient and drugs costs (β_*SD*_ = 0.276, 95% CI = 0.104, 1.181), direct non-medical costs (β_*SD*_ = 0.287, 95% CI = 0.140, 1.206), and total economic burden (β_*SD*_ = 0.311, 95% CI = 0.186, 1.262); besides, time interval between 4 and 6 months was significantly related to large indirect costs (β_*SD*_ = 0.230, 95% CI = 0.098, 1.363) in the 12-month period after diagnosis. Similarly, time interval between 1 and 3 months was significantly associated with high direct non-medical costs (β_*SD*_ = 0.198, 95% CI = 0.004, 1.013) in the 12-month period after diagnosis. However, time interval from diagnosis to treatment was not correlated to economic costs in the recent 12 months. In the long term, shortening the time interval from diagnosis to treatment would reduce the economic burden on families, especially in the 12-month period after diagnosis.

## Introduction

Autism spectrum disorder (ASD) is a highly heterogeneous group of neurodevelopmental disorders characterized by different levels of impairment in social communication and interaction, repetitive behaviors, and restricted language ability (1). An increasing prevalence of ASD has been reported worldwide regardless of ethnic or cultural background (2–4). A meta-analysis study indicated that the pooled prevalence of ASD in mainland China was 3.92‰ (5). However, the data supporting this estimation were not accurate due to regional-level information. The real number of autistic children was likely to be much higher (6).

There is a lack of unified and effective approaches for the prevention and treatment of ASD in clinical practice. The long-term prognosis of most individuals with ASD is poor, namely, they lack the ability to study and work independently in adulthood (7). Therefore, these patients have to rely on parental care for extended periods and need long-term rehabilitation, which probably leads to heavy economic burden to families and society (8, 9).

The direct and indirect economic costs are two aspects of the total economic costs of ASD. The direct costs are related to diagnosis, medical check, rehabilitation, and behavioral therapy. Referring to Rogger et al., the direct economic costs is direct economic costs are defined as the total sum of ASD-related medical or healthcare costs and therapeutic costs (10). Montes et al. estimated that raising a child with ASD costs at least twice as much as raising a typically developing child (11). Ganz et al. reported that the lifetime per-capita incremental societal cost of autism was $3.2 million in the United States (12). Using the national data from the UK, researchers have summarized that ASD was associated with $3,020 higher annual healthcare costs and $14,061 higher aggregate no-healthcare costs, including higher school costs of $8,610, compared with the costs for children without ASD. Horlin et al. reported that the median cost in families of children with ASD in Australia was approximately AUD 34,900 (13). In the present study, the administrative data on ASD-related health service use and special education costs have limited accessibility in China (14). Scholars reported ASD-related costs by self-designed questionnaire. For instance, Xiong et al. reported that the yearly rehabilitation cost for a child with autism was 19,196.72 RMB in Beijing, which was significantly higher than the cost for children with physical and mental disabilities (15). Ou et al. interviewed 59 nuclear families of children with ASD, and the mean annual costs of special education were higher than those for children with other disabilities and typically developing children (¥55,359 vs. ¥27,904 vs. ¥17,413, respectively) (16). In Heilongjiang Province, Wang et al. investigated 290 families of children with ASD from 2007 to 2009. They found that the costs of behavior therapy represented the largest proportion of health expenses. In addition, the costs of ASD resulted in a heavy economic burden for both urban and rural families. Approximately 19.9% of urban families and 38.2% of rural families have reported that the health costs of ASD exceeded their household income (14, 17).

Indirect costs, such as the informal care burden and lost parental productivity, are also important components of the economic costs (10). Previous studies have shown that the task of childcare forced parents out of employment or adjust job plans, which results in the family expenditures for the disease. Horlin et al. reported an estimated loss of the employment income of parents/caregivers of autistic children amounting to 90% of the total family costs of ASD, ~$29,200 (13). Another study found that the employment of parents was affected by their child's disorder and that 52.3% of parents adjusted their jobs due to childcare (16). Cidav et al. reported that the employment rate of mothers of children with ASD declined by 6%, and their weekly working time decreased by 7 h compared with mothers of typically developing children (18). Mothers' work time decreased markedly, i.e., by an average of 6.6 months per year, leading to a loss of 21,400 RMB by conversion, which was significantly higher than the loss experienced by fathers (2.0 months and 10,000 RMB) (19). Kamaralzamana investigated 245 parents of autistic children in Malaysia, and the study reported that the highest cost of indirect expenditures was 963.99 RM (23). A survey conducted in Chongqing city pointed out that the mean rehabilitation costs of 92 children with ASD were 3,500 RMB per month for each of them and the rehabilitation costs accounted for ~72% of the household income in those urban families (20).

Several surveys have explored the factors related to the economic costs in families of children with ASD. For instance, Parish et al. found that per-capita Medicaid expenditures were likely to reduce the financial burden in families of children with autism (21). Horlin et al. reported that different levels of ASD symptoms were significant predictors of the cost of ASD (13). Similar to a previous study, having a child with ASD significantly decreased the likelihood of living in a higher-income household family (11). Sharpe et al. reported that family income, autism symptoms, and ASD-related therapy costs were associated with financial problems in families with autistic children under the age of 19 (22). The age of children, the family income, and the combined disorder of children were related factors of direct and indirect costs (23). Callander et al. reported that autistic children's age (2–11 years) was relevant to the sum of absentee days and the dissatisfactory working status of parents (24). Mothers' education level, rural residence, and comorbidity count of children influenced the state health expenditure in families of children with autism (22).

In summary, there was more significant burden associated with ASD (on a population level) as compared to other diagnoses (such as ADHD or ODD) and children with normal development. It is very important to note that the earlier the treatment for children with ASD, the better the children's prognosis for developing language and social skills, which, to some extent, also means less ASD-related economic burden for the family. It was suggested that the time interval from the date children first diagnosed with ASD to the date of first speech/behavior treatment was strongly associated with the economic burden in families with autistic children. However, there was limited evidence to support this conclusion. Therefore, this study aimed to investigate the economic costs in families with autistic children and explore its potential association with the time interval from diagnosis to treatment. We hypothesized that the family economic costs were positively associated with the time interval from diagnosis to treatment.

## Methods

### Study Procedure

This was a cross-sectional study conducted in Changsha, Hunan Province, from March to November 2018. There are a total of 10 autism rehabilitation centers in the five main districts of Changsha. We selected four autism rehabilitation centers using the clustering random sampling method and recruited all the families (*n* = 150) of children with ASD from those centers. Autistic children were strictly defined as having an autistic disorder based on DSM-IV, while children with a pervasive developmental disorder not otherwise specified (PDD-NOS) or with Asperger syndrome were excluded from the study.

A questionnaire was designed to collect general information about the parents and children. With regard to economic costs, it is difficult to investigate the accurate ASD-related costs of families with autistic children in the whole course of the disease because of the recalling bias of parents, and there is rare special expense management system for ASD in China. Therefore, in the present study, a questionnaire including a list of ASD-related cost items was developed basing on the previous studies (10, 25). The questionnaire was finished by well-trained professionals through a face-to-face interview with autistic children's parents, which might eliminate some limitations on the strength of the data collected by self-report to some extent. Each participant (mother or father or both) was interviewed for ~20 min and confirmed related information. All the participants signed their informed consent after being fully informed of the purpose and meaning of the study. Ethical approval was provided by the Ethics Committee of Hunan Provincial Maternal and Child Health Care Hospital (EC20180318). Finally, 14 families that refused to take part in the study or did not complete the interviews were excluded. A total of 136 families with one autistic child were included in this study.

## Measures

### General Information

General characteristics of the family and children were provided by parents, including parents' age, education level, employment, family income in the last 12 months, residence, family economic assistance, and the ratio of ASD-related costs to the total family income. Besides, children's age, gender, and race/ethnicity were also recorded.

### Time Interval From Diagnosis to Treatment and Other Disorder Characteristics of Children

The characteristics of the autistic children included the course of ASD, the course of speech/behavior treatment, the time interval from diagnosis to treatment, and the lag time between the first 12 months after diagnosis and the last 12 months and scores of autism symptoms. Among them, the time interval from diagnosis to treatment of children was defined as the time interval from the date children first diagnosed with ASD to the date of first speech/behavior treatment. The scores of autism symptoms were evaluated by the Autism Behavior Checklist (ABC). The ABC was a screening tool used to evaluate the symptoms of autistic children with satisfactory reliability and validity, and it could be completed by the parents or caregivers of children (26). The ABC is a 57-item screening checklist for children older than 18 months and divided into five categories: (1) sensory, (2) relating, (3) body and object use, (4) language, and (5) social and self-help. According to the severity level, each item is rated from “1” to “4.” The screening cutoff score is 53, and the diagnosis cutoff score is 67 (27). The study referred to the Autism Diagnosis, Treatment and Rehabilitation Guidelines of China, which was issued by the Chinese Ministry of Health (28). Scores of <31, 31–66, and > 66 correspond to nearly normal, borderline, and autism behavioral symptoms, respectively. The standard was suitable for children with ASD (25).

### Family Economic Costs

We collected the ASD-related economic costs in two vital periods during the course of the disease including the 12-month period after the date of the child was first diagnosed with ASD and the most recent 12 months before the date of interview. Most of the parents would seek medical consultations around everywhere during the first period, which resulted in large costs for medical-related expenditure, while in the second period, the expenditure of ASD-related interventions and therapies in most families was relatively stable and reflects the economic burden. The parent-reported ASD-related economic costs were divided into three categories: direct, indirect, and total economic costs.

(i) Direct economic cost was calculated by the following formula:
  Y_1_ = X_1_ + X_2_ + X_3_
  X_1_: inpatient, outpatient, and drug expenses (refer to outpatient, inpatient, and drug costs for the confirmation of diagnosis at hospital and the short-term drug therapy, which reflect the costs for diagnosis and short-term drug therapy).
  X_2_: therapeutic costs (refer to speech/behavior rehabilitation costs occurring in rehabilitation centers and hospitals, which reflect long-term costs for speech/behavior intervention).
  X_3_: direct non-medical costs (refer to expenses related to illness including transportation, accommodation, and living expenses during the processes of outpatient and inpatient treatment);
(ii) Indirect economic cost was defined as production loss for parents, which was calculated by the human capital approach as follows (29):
  Y_2_ = ${\text{X}}_{4}^{*}$X_5_
  X_4_: labor force time lost by parents
  X_5_: labor-time value per unit
  Specifically, the labor-time value per unit was divided into farmer and resident labor value. In our study, the participants were composed of residents in the urban areas and a small number of farmers. The per-capita net of farmer and resident labor value in Hunan Province was collected by the Hunan Provincial Bureau of Statistics (https://tjj.hunan.gov.cn/). For example, the per-capita net income of resident labor in Hunan Province in 2018 was 25,241 RMB, so if there were 180 total days of labor time lost for an adult, the indirect economic costs would be 12,447 Yuan (180^*^25,241/365).
(iii) The total economic cost of children with ASD was calculated by the following formula:
  Y = Y_1_ + Y_2_

## Statistical Analysis

Statistical analysis was performed using SPSS version 23.0. The continuous data following the law of normal distribution were described as the mean ± SD. Otherwise, the median and quantiles (P_25_ and P_75_) were reported in the study. The categorical data were described as *n* (%). Multivariate linear regression models were carried out to explore the probable association between the family economic costs and the time interval from diagnosis to treatment after adjusted for the general characteristic of families and children. The *Z*-score transformation was used to transform the raw data pertaining to the economic costs in the 12-month period after diagnosis and the most recent 12 months to a linear, additive scale as a dependent variable in the linear models. A *p*-value of <0.05 was considered statistically significant in our study.

## Results

### General Information

Table 1 shows the general information of the parents and children. The mean age of the mothers and fathers were 30.01 (SD = 4.65) and 34.06 (SD = 4.97) years, respectively. There were 52.2 and 57.4% of mothers and fathers with a university degree, respectively. In total, 59 mothers (43.4%) were unemployed, and they reported that their jobs were more or less affected by their children's disorder. In contrast, 99.3% of fathers had a job at the time of the interviews. Sixty-two families (45.6%) reported a total family income between 20,000 and 79,999 RMB in the past 12 months. Another 42 families (30.9%) reported that their total family income was <20,000 RMB in the past 12 months; this was less than the per-capita disposable income of residents in Hunan Province in 2018 (25,241 RMB). Sixty families (44.1%) reported that their children were or had been supported with 12,000 RMB to 20,000 RMB 1-year economic aid from disabled persons' federation/public interest organizations. In total, 55 families (40.4%) reported that in the past 12 months, ASD-related costs accounted for over 50% of their total family income. Seventy families (51.5%) reported that in the past 12 months, ASD-related treatment costs had accounted for 25–50% of the total family income.

**Table 1:** General information of participants and disorder characteristics of children.

**Variables**	***n***	**%**
**Mothers' education level**		
Junior high school or less	28	20.6
High school	37	27.2
University degree	71	52.2
**Fathers' education level**		
Junior high school or less	25	18.4
High school	33	24.3
University degree	78	57.3
**Employment of mothers**		
Employed	77	56.6
Unemployed	59	43.4
**Employment of fathers**
Employed	135	99.3
Unemployed	1	0.7
**Total income of family in recent 12 months (RMB) ddmonmonthsmonths (RMB)**		
<20,000	42	30.9
20,000–79,999	62	45.6
≥80,000	32	23.5
**Whether the family received economic aid from disabled persons' federation/public interest organizations**		
Yes	60	44.1
No	76	55.9
**What percentage of the ASD-related costs account for total family income in the recent 12 months?**		
<25%	11	8.1
25–50%	70	51.5
> 50%	55	40.4
**Age of children (years)**		
<4	44	32.4
4–5	71	52.2
> 5	21	15.4
**Gender of children**
Male	99	72.8
Female	37	27.2
**Residence of family**
Urban	95	69.9
Rural	41	30.1
**Race/ethnicity of children**
Han Chinese	122	89.7
Minority Chinese	14	10.3
**The course of ASD (months)**
<21.5	68	50.0
21.5–38.5	34	25.0
> 38.5	34	25.0
**The course of speech/behavior treatment (months)**
<10	66	48.5
10–24	40	29.4
> 24	30	22.1
**Time interval from diagnosis to treatment of children (months)**		
<1	63	46.3
1–3	25	18.4
4–6	15	11.0
> 6	33	24.3
**The lag time between the first 12 months after diagnosis and the last 12 months (year)**		
≤ 1	81	59.6
> 1	55	40.4
**Score of symptoms (ABC) of children**
<31	69	50.7
31–66	31	22.8
≥ 67	36	26.5

There were 37 girls and 99 boys between 2 and 11 years old who were diagnosed with ASD by a neurologist based on the DSM-IV were recruited into the survey. More boys (72.8%) were enrolled than girls (27.2%). Among these children, 95 (69.9%) lived in urban areas. The mean age of the children was 4.44 years (SD = 1.88). Most of the children (89.7%) were of Han ethnicity.

### Time Interval From Diagnosis to Treatment and Other Disorder Characteristics of Children

The time interval from diagnosis to treatment and other disorder characteristics of children are presented in Table 1. For 46.3% of the autistic children, the time interval from the date children first diagnosed as ASD to the date of first speech/behavior treatment was <1 month; for 18.4% of them, the time interval was from 1 to 3 months; for 11.0% of them, the time interval was 4 to 6 months; and for 24.3% of them, the time interval was longer than 6 months. The mean course of the ASD and the speech/behavioral treatment were 26.77 (SD = 21.80) and 15.43 (SD = 16.46) months, respectively. More than half of the children had a lag time between the first 12 months after diagnosis and the last 12 months of <1 year. The mean ABC score of the children was 52.07 (SD = 26.7). According to the criteria of the ABC, the constituent ratios in the <31, 31 to 66, and > 66 groups were 50.7%, 22.8%, and 26.5%, respectively (Table 1). There were two reasons explained for this phenomenon: (1) usually, the Autism Behavior Checklist (ABC) was applied in the screening of ASD, not in diagnosis; and (2) their condition improved after rehabilitation treatment in hospital or rehabilitation schools.

### The Family Economic Costs in the 12-Month Period After Diagnosis

The expenditures on autistic children in the 12-month period after diagnosis are shown in Table 2. The diagnosis of ASD resulted in massive expenditures for each family in the study. Overall, the median costs of medical expenditures (inpatient services, outpatient services, and drugs) related to ASD treatment were equal to the special education/treatment expenditures (5,000.00 RMB). The median costs of the total direct economic costs of ASD (24,202.00 RMB) were higher than the indirect economic costs (379.01 RMB). The total economic costs of ASD amounted to a median of 26,502.26 RMB (Table 2).

**Table 2:** The ec onomic costs in families with autistic children in the 12-month period after diagnosis.

**Economic costs (RMB)**	**Median**	**Quartiles**		**Min**	**Max**
		**P_**25**_**	**P_**75**_**		
The direct economic costs	24,202.00	5,721.50	50,970.00	0.00	392,000.00
Inpatient, outpatient, and drugs costs	5,000.00	2,000.00	20,000.00	0.00	304,000.00
Special education (treatment) costs	5,000.00	0.00	22,690.00	0.00	96,000.00
Direct non-medical costs	800.00	80.00	3,000.00	0.00	90,000.00
The indirect economic costs (loss of productivity)	379.01	138.26	2,838.98	0.00	26,507.48
Total economic costs of ASD	26,502.26	7,250.48	54,324.36	0.00	397,738.98

### The Family Economic Costs in the Recent 12 Months

The expenditures on autistic children in the recent 12-month period are shown in Table 3. The total direct economic costs were 28,620.00 RMB. The median costs of special education/treatment of the families were higher than the costs of medical services (20,000.00 RMB vs. 600.00 RMB). The median direct non-medical costs were 2,000.00 RMB, while the median costs of the indirect economic costs and the total economic costs were 84.17 RMB and 29,411.91 RMB, respectively (Table 3).

**Table 3:** The economic costs in families with autistic children in the recent 12 months.

**Economic costs (RMB)**	**Median**	**Quartiles**		**Min**	**Max**
		**P_**25**_**	**P_**75**_**		
The direct economic costs	28,620.00	14,262.50	40,316.00	4,000.00	147,200.00
Inpatient, outpatient, and drugs costs	600.00	0.00	5,750.00	0.00	80,000.00
Special education (treatment) costs	20,000.00	7,140.00	31,680.00	400.00	96,000.00
Direct non-medical costs	2,000.00	1,000.00	3,500.00	12.00	73,200.00
The indirect economic costs (loss of productivity)	84.17	35.44	945.83	2.95	63,971.18
Total economic costs of ASD	29,411.91	14,548.46	42,190.96	4,070.88	149,007.50

### The Association Between the Family Economic Costs and the Time Interval From Diagnosis to Treatment

The multiple linear regression models were conducted to estimate the relationship between economic costs in the 12-month period after diagnosis and recent 12 months and time interval from diagnosis to treatment. After adjusting for the characteristics of parents and children, we found that the time interval from diagnosis to treatment was associated with economic costs in the 12 months after diagnosis (Table 4). Compared with the time interval shorter than 1 month, time interval over 6 months was significantly associated with high direct economic costs (β_*SD*_ = 0.308, 95% CI = 0.177, 1.254), inpatient/outpatient and drugs costs (β_*SD*_ = 0.276, 95% CI = 0.104, 1.181), direct non-medical costs (β_*SD*_ = 0.287, 95% CI = 0.140, 1.206), and total economic burden (= 0.311, 95% CI = 0.186, 1.262); besides, time interval between 4 and 6 months was significantly related to large indirect costs (β_*SD*_ = 0.230, 95% CI = 0.098, 1.363) in the 12-month period after diagnosis. Similarly, the time interval between 1 and 3 months was significantly associated with high direct non-medical costs (β_*SD*_ = 0.198, 95% CI = 0.004, 1.013) in the 12-month period after diagnosis. However, the association between economic costs in the recent 12-month period and time interval from diagnosis to treatment was not observed in this paper (Table 5).

**Table 4:** Multiple linear regression models for economic costs in the 12-month period after diagnosis and time interval from diagnosis to treatment (months).

		**The direct economic costs**		**Inpatient, outpatient, and drugs costs**		**Special education (treatment) costs**		**Direct non-medical costs**		**The indirect economic costs (loss of productivity)**		**Total economic costs of ASD**	
		**β_*SD*_**	**95% CI**	**β_*SD*_**	**95% CI**	**β_*SD*_**	**95% CI**	**β_*SD*_**	**95% CI**	**β_*SD*_**	**95% CI**	**β_*SD*_**	**95% CI**
Time interval from diagnosis to treatment (months)^a^	<1	Reference											
	1–3	0.066	−0.345, 0.683	0.055	−0.373, 0.654	−0.098	−0.796, 0.289	**0.198**	**0.004, 1.013**	0.136	−0.170, 0.870	0.076	−0.319, 0.708
	4–6	0.116	−0.257, 0.995	0.12	−0.245, 1.006	0.039	−0.536, 0.784	0.048	−0.462, 0.765	**0.230**	**0.098, 1.363**	0.131	−0.208, 1.043
	> 6	**0.308**	**0.177, 1.254**	**0.276**	**0.104, 1.181**	0.047	−0.458, 0.678	**0.287**	**0.140, 1.206**	0.183	−0.119, 0.970	**0.311**	**0.186, 1.262**

a *Adjusted for parents' age, educational level, employment, total income of family in the recent 12 months, what percentage of the ASD-related costs account for total family income in the recent 12 months, whether the family received economic aid from disabled persons' federation/public interest organizations and residence of family, as well as children's age, gender, ethnicity, duration of disorder, duration of speech/behavior treatment, lag time between the first 12 months after diagnosis and the recent 12 months, and score of symptoms*.

*^b^Bold fonts for statistics significance*.

**Table 5:** Multiple linear regression models for economic costs in the recent 12-month period and time interval from diagnosis to treatment (months).

		**The direct economic costs**		**Inpatient, outpatient, and drugs costs**		**Special education (treatment) costs**		**Direct non-medical costs**		**The indirect economic costs (loss of productivity)**		**Total economic costs of ASD**	
		**β_*SD*_**	**95% CI**	**β_*SD*_**	**95% CI**	**β_*SD*_**	**95% CI**	**β_*SD*_**	**95% CI**	**β_*SD*_**	**95% CI**	**β_*SD*_**	**95% CI**
Time interval from diagnosis to treatment (months)^a^	<1	Reference											
	1–3	0.058	−0.387, 0.683	0.108	−0.222, 0.776	−0.131	−0.866, 0.190	−0.014	−0.520, 0.449	−0.014	−0.520, 0.449	0.048	−0.409, 0.656
	4–6	0.005	−0.635, 0.667	0.118	−0.231, 0.984	−0.140	−1.087, 0.198	0.089	−0.306, 0.873	0.089	−0.306, 0.873	0.025	−0.568, 0.728
	> 6	−0.050	−0.678, 0.444	0.046	−0.417, 0.629	−0.159	−0.923, 0.183	0.110	−0.251, 0.764	0.110	−0.251, 0.764	−0.019	−0.603, 0.514

a *Adjusted for parents' age, educational level, employment, total income of family in the recent 12 months, what percentage of the ASD-related costs account for total family income in the recent 12 months, whether the family received economic aid from disabled persons' federation/public interest organizations and residence of family, as well as children's age, gender, ethnicity, duration of disorder, duration of speech/behavior treatment, lag time between the first 12 months after diagnosis and the recent 12 months, and score of symptoms*.

*^b^Bold fonts for statistics significance*.

## Discussion

The current study investigated the relationship between economic costs and time interval from diagnosis to treatment of children with ASD based on a cross-sectional study in autism treatment centers. We reported the economic costs in families of children with ASD by collecting related costs from two vital periods during the course of the disease including 12 months after diagnosis and the recent 12 months. The ASD-related costs were an economic burden for those families. Most notably, we found that a shorter time interval would reduce the risk odds of economic poverty of those families.

The present studies generally emphasized the importance of early diagnosis and intervention for symptoms reduction. However, few studies reported the data on the interval between diagnosis and intervention. Our findings illustrated that in 75% of those children, the time interval from diagnosis to treatment was <6 months. Sun et al. indicated that the mean time between diagnosis and initial intervention was 6.5 months among 69 autistic children enrolling from 19 regions from mainland China (30). Another study by Shrestha et al. reported that the interval between help-seeking to diagnosis was 29.4 months with the longest time lag of 13 years among 50 children enrolled from Nepal (31). In our study, most of the parents enrolled in the project had a high degree of education level. Parents could make a clearer decision on whether their children need effective behavior treatment based on their knowledge. Then, parents tried their best to acquire social resources for their children in the hospital and rehabilitation centers to accept intervention as soon as possible (32). Moreover, urban areas that provided more medical resources for autistic children also shortened the time interval from diagnosis to treatment.

It was unsurprising that the disorder of children damaged the economic balance of families including an increase in family household expenditure and shortened job time of parents. For example, in our study, nearly half of the families reported that the ASD-related expenditures accounted for almost 55% of their household income; this proportion was higher than that observed in another study (14%) (11) but lower than that found in two other studies (14, 33). Xiong et al. investigated 46 families with autistic children from Beijing and found that the total direct economic burden was approximately 22,964.1 RMB per year (15).

Wang et al. analyzed annual family expenditures on ASD-related health services in 178 urban families with autistic children from Heilongjiang Province, and the mean costs were 17,497.28 RMB (14). Similar to previous studies conducted in different areas of China, the study also showed that the median cost of the total economic costs in the two vital periods considered in this project was closed to 30,000 RMB, which was higher than the per-capita disposable income of residents in the Hunan Province (25,241 RMB) in 2018. Nevertheless, the difference in studies might be due to the heterogeneity of the regions, the level of productivity, medical resources, and the symptoms of children.

In addition, adjustment in job time also has important impact on economic costs. For example, in the current study, the median indirect economic cost in the recent 12 months was 379.01 RMB, which was slightly lower than that found from a survey conducted in Malaysia (~1,500 RMB) (23). Specifically, we pointed out that most mothers' employment was affected by childcare. Relatively speaking, the father is the main labor force in a family, and the mother is usually the primary caregiver of children because of the traditional concept of gender role (men are breadwinners and women are homemakers) in China. Pregnancy and lactation may further restrict the career development of women; furthermore, women's income is still lower than men's income under the same position and level of experience. Therefore, mothers leave their work more often than fathers to take care of their autistic child. Our results underlined that the rate of unemployment in ASD families was 43.4% for mothers and merely 0.7% for fathers, which was consistent with the findings of other studies (16). Besides, limited by the availability of ASD-related health resources, many parents have to take their children across the regions to receive rehabilitation therapy for ASD in the provincial capital or larger cities (6). This is more likely to aggravate the loss of working time of parents.

The present study also demonstrated that longer time interval from diagnosis to treatment was associated with heavy ASD-related economic costs of children in the 12 months after diagnosis. The course of doctor consultation and seeking appropriate institutions was an important period before long-term intervention in a rehabilitation institution. The expenditure of this course was catastrophic because it usually exceeded an acceptable percentage of consumption or household income. For parents, the course meant less work time and higher expenditure. A previous study suggested that the early diagnosis of ASD was important for improving the effectiveness of interventions in children with ASD (34). Researchers had also called for pediatricians to strive to identify and begin interventions for children with ASD sooner rather than later (35). Jacobson et al. hold the view that the early treatment would reduce overall program costs (36). Moreover, shortening the time interval from diagnosis to treatment means “buying time” for autistic children, i.e., saving time on medical consultations to have more time to spend on training to reduce symptoms of children. If the children have more chance to accept treatment in rehabilitation centers regularly, parents would be much more likely to focus on their employment and then provide economic support for their families. To be specific, parents could concentrate on their career rather than feeling exhausted by taking care of their children.

Our findings indicated that the shorter the time interval from diagnosis to treatment, the lower the economic costs. Meanwhile, previous studies reported that early diagnosis and therapy were the keys to reduce the complications. Therefore, to alleviate their economic burden as well as improve the prognosis of their children, parents should improve the recognition of the disease in order to strive for early diagnosis and therapy. Hence, improving social services is also important to shorten the time interval between diagnosis and intervention.

Estimate of ASD-related medical and intervention costs are very crucial to evaluate future care needs and resources allocation. We reported ASD-related costs in two vital periods during the course of disease, which objectively reflected the economic burden in those families and received more attention. Nonetheless, there are several limitations in this study as it is designed as a cross-sectional study with a small sample size. Namely, only one geographic area of China was examined, which might impact the generalizability of this study. Further investigations with a larger number of families with autistic children (especially involving recruitment of families in different regions of the country) will be necessary to completely rule out such limitation.

## Conclusion

The present study investigated the economic costs in families of children with ASD, and the results revealed that ASD-related costs were a heavy economic burden for those families. Shortening the time interval from diagnosis to treatment was beneficial for reducing the total economic burden to some extent. Further investigations with a larger number of subjects will be necessary in the near future to verify the present conclusion.

## Data Availability Statement

The raw data supporting the conclusions of this article will be made available by the authors, without undue reservation.

## Ethics Statement

Ethical approval was provided by the Ethics Committee of Hunan Provincial Maternal and Child Health Care Hospital (EC20180318). The patients/participants provided their written informed consent to participate in this study.

## Author Contributions

WZ, XX, and HX designed the study. WZ, KW, SC, DL, and XX collected the data. WZ, XX, and HX analyzed the results. WZ wrote the main manuscript text. XX and HX reviewed the manuscript.

## Funding

This study was financially supported by the National Natural Science Foundation of China (81701356), Major Scientific and Technological Projects for the Collaborative Prevention and Control of Birth Defects in Hunan Province (2019SK1015), the Science and Technology Programs of Changsha city (kq1801084), and Technology Projects of Administration of Traditional Chinese Medicine (201918).

## Conflict of Interest

The authors declare that the research was conducted in the absence of any commercial or financial relationships that could be construed as a potential conflict of interest.

## Publisher's Note

All claims expressed in this article are solely those of the authors and do not necessarily represent those of their affiliated organizations, or those of the publisher, the editors and the reviewers. Any product that may be evaluated in this article, or claim that may be made by its manufacturer, is not guaranteed or endorsed by the publisher.

## Abbreviations

ASD: Autism Spectrum Disorder
ABC: Autism Behavior Checklist.
